# Knowledge and perceptions of health and environmental risks related to artisanal gold mining by the artisanal miners in Burkina Faso: a cross-sectional survey

**DOI:** 10.11604/pamj.2017.27.280.12080

**Published:** 2017-08-21

**Authors:** Adama Sana, Christophe De Brouwer, Hervé Hien

**Affiliations:** 1 Centre Muraz, Bobo-Dioulasso, Burkina Faso; 2 Université Libre de Bruxelles, School of Public Health, Environmental and Occupational Health Research Center, Brussels, Belgium; 3 Institut de Recherche en Science de la Santé (IRSS/CNRST), Ouagadougou, Burkina Faso

**Keywords:** Artisanal mining, risks, perception, knowledges, Burkina Faso

## Abstract

**Introduction:**

Artisanal gold mining is an activity ensuring the survival of about 700,000 families in Burkina Faso with a considerable contribution to the national economy. Techniques and chemicals used in the operation, have adverse impacts on health and the environment. Our study aims to evaluate the perceptions and knowledge of these different impacts among artisanal gold miners.

**Methods:**

A cross-sectional survey was conducted in artisanal gold mines Bouda and Nagsene in the region of the North of Burkina Faso. Two hundred miners over 18 years of age were interviewed.

**Results:**

All the participants have recognized that gold mining has health impacts and 88.5% felt these impacts as important with a significantly higher proportion among those with more than 3 years' seniority (p = 0.001). The environmental impacts were perceived as important by 64.5% of miners, with a significant difference according to the position (p = 0.004). Sixty percent (60%) of respondents could identify at least 3 of the 5 health impacts of gold mining listed and 49.5% acknowledged at least 3 impacts on the environment. The diggers had significantly more knowledge about the symptoms (p < 0.001).

**Conclusion:**

Study highlights the lack of knowledge of the Stampeders on the health and environmental impacts of artisanal gold mining. Findings might be used to develop more effective awareness campaigns in the future. Communication with diggers must focus on the risk perception because it appears that raising risk perceptions from low to high would have a major effect on behavior.

## Introduction

Artisanal gold mining knows a craze ceaselessly increasing in several countries through the world. Artisanal and small-scale gold mining produce together about 10-15% of the world's gold [1]. The United Nation Environment Program estimated that more than 15 million people, including three million women and children, participate in more than 70 countries in this activity [2]. In Burkina Faso, there are about 200 sites across the country [3]. The exact number of artisanal gold miners in activity is difficult to determine, because it is an ephemeral activity where people come and go depending on the seasons and the discoveries of new veins [4]. However, more than 200,000 people [4, 5] participate actively in artisanal gold mining, of which 38% of women and 11% of children aged less than 15 years [6]. Eighty percent (80%) of the workers concerned come from rural areas, often without any level of education [6]. Artisanal gold mining is a source of livelihood for many families in Burkina Faso and contributes significantly to the national economy. In the region of the North of Burkina Faso, area with a strong mineral potential, this activity has experienced booming these past 20 years. This increase is firstly the fact of climate change with corollary the rainfall declines and the degradation of arable soils and secondly, the increase in the price of gold at the international level. Artisanal gold mining can represent an opportunity to transfer wealth to rural communities already proven.

However, extraction methods, concentration and recovery techniques of gold and chemicals such as mercury and cyanide used in the activity inevitably leads to adverse consequences on ecosystems, on workers and community health [7, 8]. Artisanal mining leads to changes of the landscape and the occupation of the ground, turning large tracts of land in lunar landscape with a succession of holes and piles of rejects, not conducive to animal life and where plants are struggling to push. Environmental impacts are also relevant to the quality of waters and aquatic ecosystem degradation [7]. In addition, the infiltration of toxic substances in the soil can have side effects on the quality of soils, crops and food, even over long distances [9, 10]. The presence of fine particles in the air increases the risk of lung and cardiovascular diseases. The lack of collective and individual protection measures in most stampeders (artisanal miners), potentiated the risk of contamination by the chemical components of the ore-gangue [11]. Thus, there is risk of contamination through the skin by the manipulation of gangue, risks of inhalation and/or ingestion of these chemical components. Direct exposure to elemental mercury, often used for the amalgamation of gold, causes kidney, neurological, lung and autoimmune effects [12, 13]. Once it is released to the aquatic environment, microorganisms such as phytoplankton can convert inorganic mercury into methyl mercury. Methyl mercury is fat-soluble and accumulates in fish and shellfish, constituting a special danger for the development of the child in utero and at an early age. It is estimated that, among some populations dependent on fisheries livelihoods, between 1.5 and 17 children on 1000 have a deterioration of cognitive function resulting from the consumption of fish containing methyl mercury [12]. In 2013, the United Nations Program for Development (UNDP) estimated approximately 850,000, the number of people affected by the use of mercury and cyanide in gold mining in Burkina Faso [14].

Moreover, accidents are not uncommon in that sector of activity especially in crushers and diggers [11]. Cases of asphyxiation among underground workers or death by rock fall are possible. Apart from these accidents, musculoskeletal disorders (upper and lower limbs, back), sleep disruption, headache and abnormal fatigue may be observed among artisanal miners [11]. Managing risk to protect human health and the environment is a shared responsibility between communities, companies and businesses, politics and individuals including workers [15]. The assessment of the risk and its communication are major and essential elements in the process of environmental health risk management [16]. Integrated Risk assessment and risk communication methods must consider the perception of risk. This is all the more important since different theories have posited that risk perception is a predictor of risky behaviors [17, 18]. However, several approaches of risk assessment not accurately represent the concerned individual's own estimate of risk [19]. So, many times it is the stakeholder´s or the experts view on a risk that guide risk management strategies. However, several studies have shown that each person probably perceives risk differently [20, 21]. The perception of risk may vary with age, gender, and culture or education level [20–22]. Research also suggest that there is a fundamental link between the risk perception, the nature of the risk, the demographic background of the person perceiving the risk, and the social context in which the risk occurs [23–25]. Difference of risk perception may be more important when comparing the perception of the experts with that of the lay public [20, 26, 27]. In the light of these differences, policy should not, all the time, be shaped only by experts´ opinions [27]. When local conceptualization of the problem is not well understood, this can negatively influence the efficiency of risk management strategies [**19**, **20**] such as artisanal gold mining environmental health risk management.

Thus, actions aimed at reducing the risks associated with gold mining, whether purpose of information, awareness raising or attitude change must consider the perceptual dimensions of the problem at the local level. Data or studies that can provide guidance for communicating with the miners and help inform in a more appropriate process are critical, including miner's perception and knowledge researches. To our knowledge, if environmental and health risks of artisanal gold mining are known through studies operating in Burkina Faso and elsewhere [6, 11, 28, 29], the perception of the main actors such as miners and their level of knowledge about these risks need to be explored. This study is designed to assess the knowledge and identify the perception of the stampeders on health and environmental risks related to artisanal gold. The aim is to provide accurate information to policy-makers for implementation of risks management strategies, especially risks communication, in a manner that miners can understand and evaluate.

## Methods

**Scope of the study:** The study was conducted at two villages of the municipality of Yako, located in the North Region of Burkina Faso. The choice is explained by the high intensity of artisanal gold mining in this part of the country and easier access in rainy season.

**Type of study and sampling:** A cross-sectional survey was conducted in artisanal gold mines Bouda and Nagsene, in the municipality of Yako. The stampeders of at least 18 years were selected. Were included in the study, the stampeders with a seniority of at least 6 months and consenting. Because of absence of an exhaustive list of stampeders working on these sites, selection by simple random sampling proved impossible. A quotas sampling according to sex (1/3 of women, 2/3 of men) [6] was performed taking into account the fact that approximately one third of women work in the artisanal sites. To compensate for the selection bias associated with this type of sampling, the estimated size of the sample (n = 96) has been doubled to 192 subjects. A total sample of 200 subjects was considered to cover potential non-respondents. The power of the target study was at least 80%, sufficient in the event where 40.6% of the Stampeders are unaware of the health effects related to the use of mercury [30].

**Data collection:** individual interviews in face to face has been made, based on an anonymous and standardized questionnaire. The questionnaire included: socio-demographic characteristics, motivations to the practice of gold mining, the perception degree of the health and environmental impacts of gold mining, knowledge of the health and environmental impacts of gold mining, behavior in the use of protective equipment, therapeutic remedies and the need for awareness. Our study included two dependent variables that are the perception of health impact of artisanal gold mining, the perception of environmental impacts and two others concerning the knowledge of these impacts. To access the perception of risks, environmental and health impacts of artisanal gold mining were rated on a risk scale using questions asking respondents to rate: “very important”, “fairly important”, “little important”, “not important” the degree of consideration given to these risks separately. The term “important” should be interpreted as the degree of consideration given by minors to health or environmental risks of the artisanal gold exploitation (respondent judgment can be in relation to number, prevalence or severity of the concerned risk - a fuller description on these aspects was not provided to respondents). For the assessment of knowledge, 5 fair assertions to check were listed based on data from the literature. The assertions concerning symptoms that can be attributed to the panning for gold were:“cough”,“weight loss”,“musculoskeletal pain”, “heart disease”, “asphyxia or suffocation”. As for environmental impacts, the knowledge was assessed by the following responses: “deforestation”, “land degradation”, “air pollution”, “the landscape deterioration”, ”disappearing of animals”. The questionnaire was subject to empirical assessment by public health professionals from the School of Public Health of the Free University of Brussels. It has, subsequently, been translated into Moore, primary local language spoken by the stampeders in the area of study. A pre-test was conducted before the start of the investigation with 5 stampeders contacted in Ouagadougou. The survey was conducted from 23 to 26 June 2014 by 2 investigators trained for this purpose. It was completed by 4 focus-group with the stampeders dealing mainly with the process of exploitation of gold. An authorization to investigate was previously requested and obtained from the economic police responsible for the management of the two study sites. A verbal consent is requested of the miner before administration of the questionnaire.

**Analysis of the data :** The results of the survey have been encoded using Epi-Info, version 3.5.3. Description of the sample and the analysis were also performed using Epi-Info. We used conventional statistics to describe the sample. Pearson chi-square is used to verify possible association between sociodemographic variables and the variables of interest, such as perception and level of knowledge of health and environmental impacts of gold mining. In the case of non-applicability of the test of Pearson chi-square (the number of expected < 5), Fischer exact test is used. A p value < 0.05 is significant. To be able to interpret in a suitable way the result of the survey, some variables have been dichotomized as follows: the study population was divided into diggers and non-diggers. Non-diggers are composed of carriers, crushers, millers, scrubbers, tumbler and buyers; seniority in the activity with for threshold 3 years [11], theoretically significant to have a good knowledge of the risks of the activity. The threshold of 3 years represents the time after which many artisanal miners chose to remain or to leave this activity; the level of education has been divided into two categories: “no”, for those who have never been to school, and ´yes´, for those with primary, lower secondary or upper secondary education level; the scale of perception in ´important´ for very and fairly important and ´not important´ for little or not important. People who answered “Do not know”were removed in later analyzes; about level of knowledge, we used the threshold of 3 correct answers. It helped to objectify knowledge according to the different socio-demographic parameters.

**Ethical approval:** The study received the approval of the Ethics Committee of Erasme University Hospital-ULB (Brussels, Belgium), reference: P2014/174. Regarding anonymous data as defined in the belgian Royal Decree of February 2001 « Arrêté royal portant exécution de la loi du 8 décembre 1992 relative à la protection de la vie privée à l´égard des traitements de données à caractère personnel », no written informed consent was required. The ethical committee has thus waived the need for written informed consent from the participant. “All procedures performed in studies involving human participants were in accordance with the ethical standards of the institutional and/or national research committee and with the 1964 Helsinki declaration and its later amendments or comparable ethical standards.”

## Results

**Study population characteristics:** 200 artisanal miners were included in the study. Socio-demographic characteristics of participants are described in Table 1. The average age of the sample studied is 24.5 years. The sample consists of 60% (n=120) of non-diggers which 62% (n=74) are women. The self-employed represent 56.5% of respondents, 42% are employees and 1.5% are employers. The average monthly income was 25,000 CFA Francs with a minimum of 2 000 CFA and a maximum of 500,000 CFA.

**Table1:** Characteristics of the sample 2014 (N = 200)

Variables		Proportion %
**Age (years)**	**Median (min - max)**	24.5 (18-83)
**Sex**	Men	63.0%
**Marital status**	Lives alone	42.5%
	In a relationship	42.5%
**Level of education**	None	67.5%
	Primary	08,0%
	Lower secondary	22.0%
	Upper secondary	02,5%
**Duration of activity (in years)**	< 3	39.0%
	≥ 3	61.0%
**Tobacco**	Yes	Men (42.9%)
		Women (00.0%)
**Alcohol**	Yes	Men (16.0%)
		Women (04.14%)
**Locality of origin**	Yako	70.0%
**Ethnic group**	Mossi	94.0%
**Post on the site**	Diggers	40.0%
	Non-diggers	60.0%

**Perception of the health impacts of gold mining:** All participants were unanimous that artisanal gold mining has health impacts. However, 88.5% of the study participants considered the health impacts of artisanal gold mining to be important (Table 2) The proportion of subjects with emphasis to health risks was significantly higher in those with a seniority of at least 3 years in activity (p = 0. 001) (Table 3). The frequency of the stampeders giving importance to the health effect of the artisanal gold mining effects seems to vary nor with the position, nor with the study level (Table 3). The environmental impacts of the activity were perceived as important by 64.5% of the Stampeders with a significant difference according to post (p = 0, 004) (Table 4). There is a non-significant difference of perception with respect to sex.

**Table 2:** Scale of perception of the health and environmental impacts of artisanal gold mining by the stampeders of Bouda and Nagsene artisanal gold mines in Burkina Faso (N = 200), 2014

Perception	Health impacts		Environmental impacts	
	Respondents (n)	Proportion (%)	Respondents (n)	Proportion (%)
**Important**	177	88.5	129	64.5
**Not important**	21	10.5	69	34.5
**Do not know**	2	1.0	2	1.0
**Total**	200	100	200	100

**Table 3:** Factors related to the perception of the health impacts of artisanal gold mining 2014 (N = 198)

Variables	Respondents (n)	Perception of the importance of the health impacts of artisanal gold mining		Χ^2^	OR (95% CI)	P-Value
		Important (%)	Not important (%)			
**Sex**						
Male	125	90.4	9.6	0.36	1.32 [0.53 - 3.31]	NS
Female	73	87.7	12.3			
**Post**						
Non-Diggers	79	88.6	11.4	0.09	0.87 [0.35 - 2.18]	NS
diggers	119	89.9	10.1			
**Studies**						
Yes	65	84.6	15.4	2.33	0.50 [0.20 - 1.27]	NS
Non	133	91.7	8.3			
**Duration of activity**						
< 3 years	77	80.5	19.5	10.47	0.22 [0.08 - 0.58]	0.001
≥3 years	121	95	5			

**Table 4:** Factors related to the perception of the environmental impacts of artisanal gold mining (N = 198) 2014

Variables Number	Respondents (n)	Perception of the importance of the environmental impacts of artisanal gold mining		Χ ^2^	OR (95% CI)	P-Value
		Important (%)	Not important (%)			
**Sex**						
Male	125	60.8	39.2	2.83	0.58 [0.31 - 1.10]	0.093
Female	73	72.6	27.4			
**Post**						
Diggers	79	53.2	46.8	8.32	0.42 [0.23 - 0.76]	0.004
Non-diggers	119	73.1	26.9			
**Studies**						
Yes	65	67.7	32.3	0.27	1.18 [0.63 - 2.22]	NS
Non	133	63.9	36.1			
**Duration of activity**						
< 3 years	77	66.2	33.8	0.06	1.08 [0.59 - 1.97]	NS
≥3 years	121	64.5	35.5			

**Knowledge of the health impacts of artisanal gold mining:** Sixty percent (60%) of respondents acknowledged at least 3 of the 5 health impacts of artisanal gold mining, listed. Analyses reveal a statistically significant association between the post and the level of knowledge of the symptoms that may be linked to activity (p < 0.001). Indeed, the proportion of participants having been able to identify at least 3 symptoms attributable to the artisanal gold mining is higher in diggers (75%) than non-diggers (50%) (Table 5).

**Table 5:** Knowledge of symptoms related to artisanal gold mining according to socio demographic characteristics of the stampeders in artisanal gold mines Bouda and Nagsene in Burkina Faso, 2014 (N = 200)

Variables	Respondents (n)	Number of symptoms related to artisanal gold mining identified		Χ^2^	OR (95% CI)	P-Value
		< 3 (%)	≥3 (%)			
**Sex**						
Male	126	40.5	59.5	0.03	1.05 [0.59 - 1.90]	NS
Female	74	39.2	60.8			
**Post**						
Diggers	80	25	75	12.50	0.33 [0.18 - 0.62]	0.000
Non-diggers	120	50	50			
**studies**						
Yes	65	33.9	66.1	1.52	0.68 [0.36 - 1.26]	NS
Non	135	43	57			
**Period of activity**						
< 3 years	78	41	59	0.06	1.07 [0.60 - 1.91]	NS
≥3 years	122	39.3	60.7			

**Knowledge of the environmental impacts of artisanal gold mining:** At least 3 of the 5 environmental impacts of gold mining in the list have been identified by 49.5% of respondents. The diggers had significantly more knowledge about environmental impacts than non-diggers (p = 0. 015) (Table 6).

**Table 6:** Knowledge of the environmental impacts associated with artisanal gold mining according to socio-demographical characteristics of the stampeders in artisanal gold mines Bouda and Nagsene in Burkina Faso, 2014 (N = 200)

Variables	Respondents (n)	Number of environmental impacts related to gold mining, identified		Χ^2^	OR (95% CI)	P-Value
		< 3 (%)	≥ 3 (%)			
**Sex**						
Male	126	52.4	47.6	0.48	1.23 [0.69 - 2.18]	NS
Female	74	47.3	52.7			
**Post**						
Diggers	80	40	60	5.88	0.49 [0.28 - 0.88]	0.015
Non-diggers	120	57.5	42.5			
**Studies**						
Yes	65	44.6	55.4	1.33	0.70 [0.39 - 1.28]	NS
Non	135	53.3	46.7			
**Period of activity**						
< 3 years	78	50	50	0.01	0.97 [0.55 - 1.71]	NS
≥3 years	122	50.8	49.2			

**Prevention behavior:** In the studied group, 7.5% of subjects reported the use of personal protective equipment (PPE) including gloves, dust masks and goggles in respectively 4,5%, 2.5% and 0.5% of cases. Ninety-nine percent (99%) of the subjects reported having ever received information to make them aware of the environmental and health risk of artisanal gold mining .98.5% think that awareness campaigns are necessary.

## Discussion

Before the discussion, it should be noted that one of the major limitations of this study is the type of sampling used. Because of absence of a list of the artisanal gold miners, we opted for a non-random sampling. However, to make up for the selection bias associated with this type of sampling, the estimated sample size has been doubled to 200 subjects. Moreover, quantitative risk assessment does not necessarily address threats to health as the community perceives them. The ideal way would be to combine the data provided by the structured methodology of this study with appropriate qualitative approaches. Additionally, the informal nature of the activity, does not allow for participants to be truthful which can go to wrong conclusion. The conclusions drawn from this study cannot be generalized at the national level, or in other populations, to reflect the perception and knowledge of the artisanal miners on the health and environmental impacts of artisanal gold mining, as the situation might be different on sites having already benefited from education or health promotion interventions. This result of study of the miners' risks perception at the time of the survey, may have different result in another time with same approach of study. Our investigation found that most participants in the study are young people with little or no education, come from rural area, and have a seniority of at least three years in the activity of gold mining. More than 4/5th of the respondents give a great importance to the impact of gold mining on health among the oldest in the activity. On the significance of environmental impacts, it is less perceived, especially by the diggers. However, miners seem to be better acquainted with the health impacts of the artisanal gold exploitation compared to the environmental impacts. The diggers are those who best know the environmental impacts of the activity.

In accordance with the analysis of our focus group results, perception of the Stampeders against the health risks of their activity, seems to be determined by the perceptions that these individuals have of disease. The perception of the health impacts of artisanal gold mining is not comparable to a perception survey about their health, although they tend to report the perception they have of health impacts of gold mining in the way they see their health as a practitioner of this activity. This is the case of a hair of ore which stated, in response to the question “do you think that gold mining can have impacts on health?”: “much even! Look at the work we do. Can you do it without getting sick? Dust gives us the cough, cold, physical effort gives us pain in the heart and the evening was sore.” So, we can equate this emphasis on the health impacts of mining to a poor perception of participants about their state of health. It was also shown that risk that threaten the life or physical integrity of the individual or someone close to him or her is associated with increased concern about those risks [20]. Miners with longer experience seem more aware of the importance of the health risks of artisanal gold mining. Through long-time observation and practice, they have accumulated a rich body of knowledge regarding artisanal gold mining risks and become more aware of the risks the longer they spend doing the job. It seems to us that their perception is due to the duration in the activity that give opportunity to experience cases of illness as workers in the mining of ore. Personal experience is known to be very important in risk perception [31–33]. For example, studies on climate change have shown that people who have had direct experience of flooding are more likely to accept climate change as serious risk [34, 35]. Part of this difference between the two levels of seniority may be the result of the difference of age between these two groups. Additional analysis found that the mean age of workers with less than 1 year seniority or between 1-2 years or 3-5 seniority (respectively 21.81, 24.10 and 23.57) is significantly low than those with more than 5 years of service (33.67 years), P value < 0.0001 in all the cases. Older people feel more vulnerable to health risks. For Bianco, the older subjects, because of their experience, were significantly more likely to be knowledgeable on the effects of environmental risks [21].

Our study doesn't show significant difference in perception between men and women, diggers compared to non-diggers. Contrary to our results, some studies showed differences in perceived risk by gender, age, education and location [36, 37]. The Institute of research and documentation in health economics (IRDES), in a survey conducted among immigrants in France suggests that the probability of declaring a bad state of health declines significantly with the level of education and social status and that women report more ill health than men [38]. Our finding could be explained by the homogeneity of the sample, all participants working in the same sector of the gold panning with almost similar working conditions [39, 40]. Even if the finding is not statistically significant, the difference in perception related to the education level (subjects without schooling are more concerned (P= 0.127)) suggests that perception is not only the fact of knowledge. In reality, no general conclusion was reached on the differences in the level of concern about risks of people with different levels of educational qualifications [34, 41]. In some studies, higher education level of the greater risk perception [41, 42], while in others the higher education groups appeared to care less [43, 44]. It is a complex association that can be influenced by several factors and therefore be variable across populations [41]. Finding that miners place less importance on environmental effects of gold mining compared to health-related perception is not surprising. Wang in his study on farmer's perception concerning biomass supply activities risks found that, “when risk perceptions were divided into personal- and environment-related perceptions, most of the respondent expressed concern for the personal risks” [45]. Even considering the cultural meaning of environment, any of these persons have an anthropocentric vision of ecosystem. This cultural perception of the environment could induce bad management practices involving a land degradation [46] and artisanal gold mining which environmental effects are becoming increasingly visible. Moreover, the fact that these impacts do not affect physical integrity can explain this result [20].

Although not reaching significance (p = 0.09), females tended to be more concerned about environmental issues. This has been reported also in many studies [42, 43, 47]. It seems that those who both cause the most visible impact to the environment and are in touch with them, are those who attach least importance to these impacts, as shown the significant difference between diggers and non-diggers. Even though, this does not mean they are ignorant of environmental problems (pollution of water, air or soil degradation) as caused by the activity. By being always in touch with these impacts, they become familiar to them (even banal), consequently these workers on the one hand have the detailed knowledge of effects and on the other hand they appear to care less [20, 41, 48, 49], give consideration first to financial benefit and health-related effects. Conventional family obligations require men (especially fathers) to be the bread-winners (providing financial support) as the head of the family [41]. As all diggers are men compared to non-diggers composed of 62% women, this can partly explain why diggers were less concerned by environmental risks. The understanding of the health impacts compared to the knowledge of the environmental impacts of gold mining could be explained by the fact that the health effects seem more obvious: history of related diseases activity, frequency of some symptoms attributable to gold panning, which is not the case of the environmental impacts, especially for non-diggers. The significant difference of knowledge between diggers and non-diggers is the proof. The diggers are those who participate and alongside the most visible environmental impacts of the activity such as degradation of the soil (well), the disappearance of the animal, the impact on the vegetation. According to Weiss, the diagnosis is based on both personal experience and a set of characteristics of the environment [50]. Even with the difference of knowledge and perception between men and women, diggers and non-diggers, environmental compared to health-related consequences, miners' still have a low awareness of environmental protection and conservation.

Despite the explosion of the activities of artisanal gold mining, the programs of awareness and health education focusing on the risks of the activity, are lacking [30]. Only 1% of the stampeders have received sensitization on the health risks associated with gold mining. Nevertheless, the impact of awareness campaigns remains questionable. Indeed, knowledges and awareness are often no influence over the way we act and the decisions we take regarding avoidance, control or protection against exposure [30]. Conversely, some authors suggest that knowledge of environmental risk and perception of this as a serious health hazard are predictors of intentions to act and implement reduction procedures [17, 25]. Because of the low rate of stampeders who reported having benefited from a campaign in our sample, we could not study the impact of raising awareness on perception, behavior or knowledge of risks. In a context characterized by material deprivation, artisanal gold mining becomes a necessity, even a survival activity. Artisanal miners focus more on their economic situation. Nevertheless, we still optimistic that participatory awareness campaigns that would involve artisanal miners will make them understand the potentially serious consequences of long-term environmental- and health-related risk of artisanal gold mining; and then they will likely learn to take appropriate preventive measures. Widespread consultation therefore plays an important role in effective risk management. For effective risk management, the contributions of the experts, stakeholders, and members of the affected public is essential for mutual understanding and development of constructive decision-making [20] This study has provided a model of artisanal miners' risk perceptions on gold mining activity. The findings provide evidence for the influence of economic factors, social factors such gender and educational differences on miners' perceptions. Our study highlight a lack of knowledge and awareness about environmental and health effects of artisanal gold mining. Findings might be used to develop more effective awareness campaigns for use in the future, especially on non-diggers who, according to the findings, have lower levels of knowledge about the environmental risks of mining. Communication with diggers must focus on the risk perception because it appears that raising risk perceptions from low to high would have a major effect on behavior [17]. Globally, it seems like these diggers want to tell us this: we dig, we chase animals away their habitats, we cut trees, pollute water, we know all of that; but all these elements were not more important than our own or our family survival! How can't we understand that, if we know they put their health and own lives in danger in the holes of ore (they perceive it) for this same survival! What could be more important than survival? This is a question, with as much importance as his response. We think that awareness raising campaigns and efficient communication strategy targeting miners should contain a statement answering this question. In this last category, participatory approach will help to build trust between miners and policy-makers.

## Conclusion

The findings provide evidence for the influence of economic factors, social factors such gender and educational differences on miners' perceptions. Our study highlight a lack of knowledge and awareness about environmental and health effects of artisanal gold mining. Findings might be used to develop more effective awareness campaigns for use in the future, especially on non-diggers who, according to this study, have lower levels of knowledge about the environmental risks of mining. Communication with diggers must focus on the risk perception because it appears that raising risk perceptions from low to high would have a major effect on behavior.

### What is known about this topic

- Artisanal gold mining is an activity involving more than 15 million people, including women and children;
- The use of chemical substances such mercury for gold recovery may lead to neurological, kidney, lung and auto-immune effects;
- Research suggest that there is a link between risk perception, the nature of the risk, the demographic background of the person perceiving the risk and the social context that the risk occurs . It also appears that raising risk perceptions from low to high would have a major effect on behavior.

### What this study adds

- The findings provide evidence for the influence of economic factors, social factors such gender educational differences, position (diggers or no-diggers) and seniority on miners' risk perception;
- Our study highlights a lack of knowledge and awareness about environmental and health effects of artisanal gold mining;
- Miners' seem to be better acquainted with the health impacts of the artisanal gold exploitation compared to environmental impacts.

## Competing interests

Authors declared they have no conflict of interest.
